# Advancements in Plant-Derived sRNAs Therapeutics: Classification, Delivery Strategies, and Therapeutic Applications

**DOI:** 10.3390/ijms26094277

**Published:** 2025-04-30

**Authors:** Qianru Rao, Hua Hua, Junning Zhao

**Affiliations:** 1 School of Pharmacy, Chengdu University of Traditional Chinese Medicine, Chengdu 611137, China; 2 Translational Chinese Medicine Key Laboratory of Sichuan Province, Sichuan Institute for Translational Chinese Medicine, Chengdu 610041, China; 3 National Center for Nanoscience and Technology, Beijing 100190, China

**Keywords:** plant-derived small nucleic acid, classification, delivery strategies, therapeutic activities

## Abstract

Plant-derived small RNAs (sRNAs) have garnered significant attention in nucleic acid therapeutics, driven by their distinctive cross-kingdom regulatory capabilities and extensive therapeutic promise. These sRNAs exhibit a wide range of pharmacological effects, including pulmonary protection, antiviral, anti-inflammatory, and antitumor activities, underscoring their substantial potential for clinical translation. A key advantage lies in their delivery, facilitated by plant-specific nanovesicular carriers—such as plant exosomes, herbal decoctosomes, and bencaosomes—which protect sRNAs from gastrointestinal degradation and enable precise, tissue-specific targeting. This review provides a comprehensive analysis of plant-derived sRNAs, detailing their classification, gene-silencing mechanisms, and nanovesicle-mediated cross-kingdom delivery strategies. It further explores their therapeutic potential and underlying molecular mechanisms in major human diseases. Additionally, we critically evaluate current technical challenges and propose future directions to advance the development of plant-derived sRNAs for precision therapeutics. This work aims to offer a robust theoretical framework and practical guidance for the clinical advancement of plant-derived sRNA-based therapies.

## 1. Introduction

Plant-derived small RNAs (sRNAs) have recently emerged as a focal point in nucleic acid therapeutics, owing to their unique gene-regulatory functions and substantial therapeutic potential. These sRNAs exert their biological effects primarily through sequence-specific recognition, enabling complementary binding to the 3′ untranslated region (3′ UTRs) of target mRNAs, which triggers degradation or translational repression via the RNA-induced silencing complex (RISC) [1,2]. In this process, sRNAs guide the RISC to selectively recognize and bind target gene sequences, facilitating precise gene regulation [3]. Notably, various plant species are rich in functional sRNAs, including microRNAs (miRNAs), small interfering RNAs (siRNAs), and synthesized oligonucleotides [4,5,6]. These sRNAs exhibit highly conserved sequence characteristics and significant homology with mammalian target genes. For instance, rice-derived MIR168a specifically binds to human low-density lipoprotein receptor adapter protein 1 (*LDLRAP1*) mRNA through complementary base pairing, highlighting remarkable cross-kingdom regulatory potential [7]. These attributes position plant-derived sRNAs as promising candidates for the development of novel nucleic acid therapeutics, offering innovative strategies for the treatment of diverse human diseases.

Plant-derived sRNAs demonstrate significant therapeutic potential, with their bioactivity validated in diverse in vitro and in vivo disease models. In pulmonary diseases, *Scutellaria barbata*-derived sRNA (BZL-sRNA-20) and *Scutellaria baicalensis*-derived sRNA (HQi-sRNA-2) exhibit pronounced immunomodulatory effects, suppressing lung cancer cell proliferation and mitigating lung injury in acute respiratory distress syndrome (ARDS) models [6,8]. In antiviral applications, honeysuckle-derived miRNA, miR2911, displays broad-spectrum antiviral activity, with demonstrated inhibitory effects against multiple viral strains, including SARS-CoV-2, H1N1, H5N1, and H7N9 [9]. For metabolic disorders, *Prunella vulgaris* L.-derived sRNA, XKC-sRNA-h3, modulates angiotensin-converting enzyme (ACE) expression across species, eliciting antihypertensive effects [10]. Similarly, *Gynostemma pentaphyllum*-derived sRNA, JGL-sRNA-h7, regulates glucose metabolism, presenting a promising novel therapeutic strategy for diabetes [11]. Despite these advances, clinical applications face challenges, including enhancing in vivo targeted delivery, ensuring biological stability, and achieving precise specificity of action. Overcoming these hurdles is critical for the successful development of plant-derived sRNA-based therapeutics.

Plant-derived exosomes and their lipid nanoparticles-mediated delivery strategies are among the extensively studied and widely employed approaches. Researchers have demonstrated that plant-derived exosomes effectively protect plant-derived sRNAs from nuclease degradation while facilitating their transmembrane transport [12]. Moreover, plant-derived sRNAs, when administrated orally, can be absorbed by gastric gland cells via the SID-1 transmembrane family member 1 (SIDT1) and subsequently encapsulated in microvesicles (MVs). These MVs facilitate intercellular communication, a process that can be visualized using confocal microscopy after fluorescent labeling [13,14]. These biological carriers offer high biocompatibility, low immunogenicity, and the ability to preserve sRNA functional integrity in complex physiological environments, while mediating information exchange via intercellular communication [12]. However, several challenges remain to be addressed, notably the plant-derived exosome isolation and purification processes, which may lead to batch-to-batch variability. Additionally, the varying physicochemical properties of exosomes derived from different plant sources may hinder their scalability for large-scale applications.

Therefore, this review systematically analyzes the classification of plant-derived sRNAs and their gene silencing mechanisms, focusing on nanovesicle-mediated cross-kingdom delivery strategies. It also explores the therapeutic potential and molecular mechanisms of sRNAs in human diseases. Notably, plant-derived sRNAs offer distinct advantages, including high target specificity, broad targeting capacity, low toxicity, and improved patient compliance. Collectively, these findings construct a robust theoretical framework for plant nanovesicle-delivered sRNA therapeutics and advancing their application in precision medicine.

## 2. The Classification of Plant-Derived sRNAs

### 2.1. Plant miRNA

**Figure 1:**
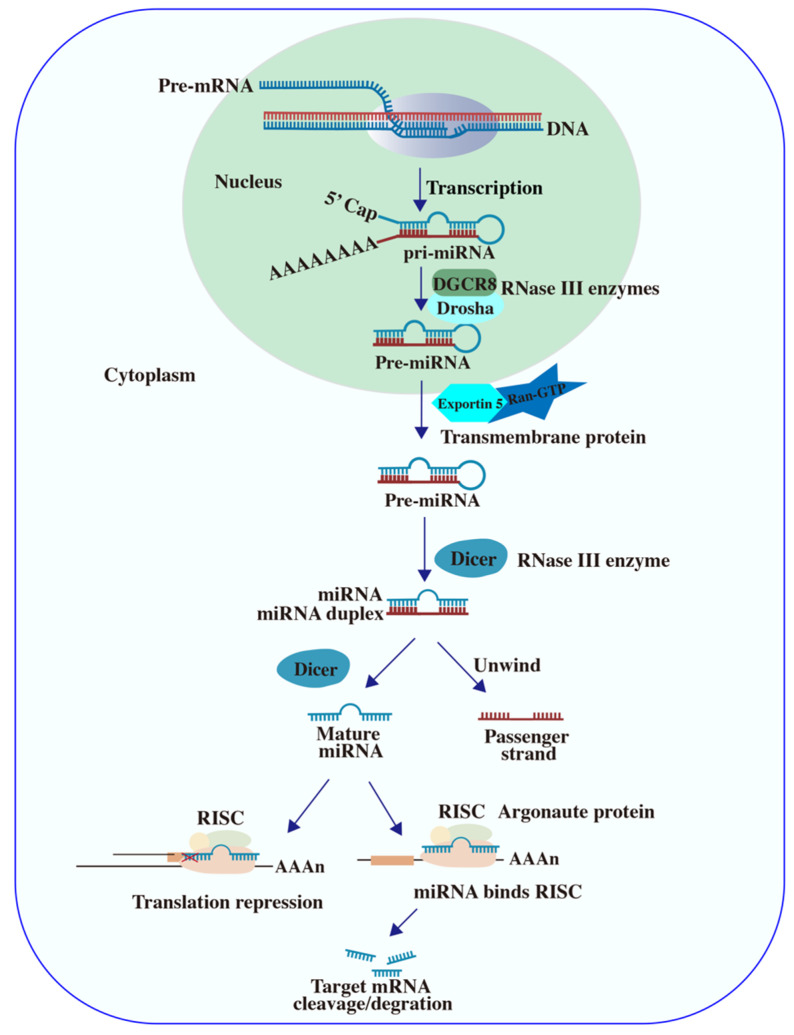
Schematic illustrations of miRNA biogenesis and function. Diagram depicting the biogenesis of miRNAs and their role in post-transcriptional regulation. Pri-miRNAs are processed into mature miRNAs, which are incorporated into the RISC complex. Mature miRNAs mediate degradation or translational repression of target mRNAs via base-pairing with complementary sequences in the 3′ UTR.

miRNAs are non-coding RNAs, typically 19–24 nucleotides in length, originating from precursor hairpin structures (Figure 1). These sRNAs mediate post-transcriptional silencing of approximately 30% of protein-coding genes by binding to complementary sequences in the 3′ UTRs of target mRNA. In the nucleus, miRNAs are transcribed as primary miRNAs (pri-miRNAs) and cleaved by the Drosha enzyme into precursor miRNAs (pre-miRNAs). Subsequently, pre-miRNAs are exported to the cytoplasm via Exportin-5 and undergo further processing by RNase III enzymes to yield mature miRNAs. These mature miRNAs associate with the RISC and the Argonaute proteins, enabling target mRNA recognition through base-pairing. This interaction typically results in mRNA degradation or translational repression, playing a critical role in post-transcriptional regulation [15,16,17,18].

Factually, miRNAs in mammals and plants exhibit distinct structural and functional characteristics. In mammals, endogenous miRNAs typically bind to the 3′ UTRs of target mRNAs through imperfect base-pairing mediated by their seed sequences (2–8 nucleotides). This interaction tolerates mismatches and bulges, resulting in lower binding affinity and increased susceptibility to degradation, partly due to the 2′-OH group acting as a nucleophile to cleave the RNA backbone [19,20]. In contrast, plant miRNAs are stabilized by methylation at the 3′ terminal nucleotide, enhancing their resistance to serum degradation and preserving their biological activity [21,22]. This stability enables plant-derived miRNAs to enter mammalian circulation and tissues via dietary intake, potentially facilitated by vesicle encapsulation or methylation modifications [21]. For instance, Zhang et al. demonstrated that rice-derived MIR168a resists gastrointestinal digestion in mammals and targets the 3′ UTR of *LDLRAP1* mRNA in liver, inhibiting its expression [23]. Additionally, mammalian cells selectively package miRNAs into extracellular vesicles in response to stimuli, releasing them into plasma or tissues [24]. Consequently, plant-derived exogenous miRNAs may modulate gene expression in recipient mammalian cells, influencing their biological functions.

### 2.2. Plant siRNA

**Figure 2:**
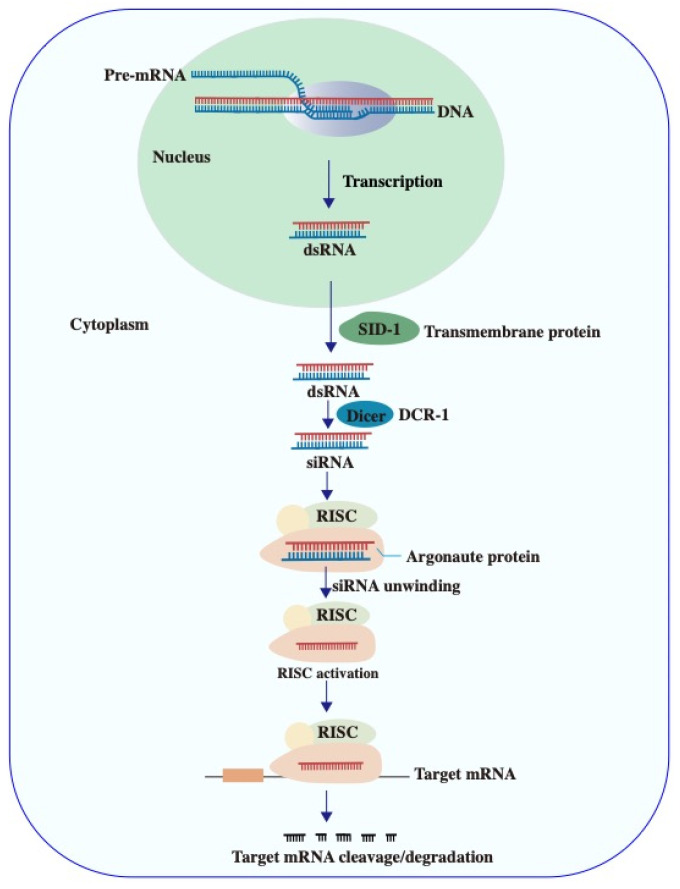
Schematic illustration of siRNA biogenesis and RNA interference. The mechanism depicts the processing of longer double-stranded RNA (dsRNA) by the Dicer enzyme into siRNAs, their incorporation into the RISC complex, and subsequent mRNA degradation or translational repression, leading to sequence-specific gene silencing.

siRNA is derived from longer double-stranded RNA (dsRNA), which is transported to the cytoplasm via the SID-1 transmembrane protein (Figure 2) [25,26]. In the cytoplasm, dsRNA is cleaved by the Dicer enzyme into shorter, double-stranded siRNAs [27]. Following cellular uptake, the guide strand of siRNA is incorporated into the RISC [28]. Once activated, RISC-bound siRNA targets precursor or mature mRNA, initiating RNA interference (RNAi) process [25,29]. This process involves the recognition and degradation of complementary mRNA, leading to sequence-specific gene silencing by inhibiting target gene expression and preventing translation of the corresponding protein, thereby eliciting therapeutic effects. However, siRNA faces challenges in vivo, including rapid degradation, facile clearance, high immunogenicity, and poor cell membrane permeability [30]. To address these limitations and enhance stability, cellular internalization, and target affinity, various delivery strategies have been developed. These include ligand-modified siRNA conjugates, lipid nanoparticles (LNPs), adeno-associated virus (AAV) vectors, N-acetylgalactosamine (GalNAc)-siRNA conjugates, and biomimetic carriers, all of which have proven effective for siRNA delivery in vivo and in vitro [31,32,33,34].

Plant-derived siRNAs are generated from both exogenous and endogenous dsRNA, regulating plant genomic expression to enhance defense against pathogens and maintaining genome stability [35]. Exogenous dsRNA in plants originate from external environment such as viral infection, ultraviolet radiation exposure, and artificially designed synthetic constructs [36,37,38]. These dsRNAs are processed into siRNAs through interactions between the plant and its environment, which then mediate RNAi process and induce target gene silences. Specifically, two tomato-derived siRNAs (siR3 and siR14) were induced in tomato leaves following infection with *Botrytis cinerea*. These siRNAs downregulate the expression of virulence genes in *B. cinerea*, thereby suppressing its virulence and inhibiting spore germination as part of the plant’s defense against the pathogen [39]. These findings highlight the potential of plant-derived siRNAs for innovative pathogen control strategies.

### 2.3. Plant Oligonucleotide

Plant oligonucleotide, encompassing naturally occurring or artificially synthesized small RNA or DNA fragments, have diverse applications, including miRNAs, siRNAs, other types of sRNAs, and artificially designed nucleotide sequences [40]. Recently, plant sRNAs have been explored for cross-species communications between cells and organisms [41]. Du et al. [42] discovered that plant-derived sRNAs can self-assemble with plant lipids to form lipid nanoparticles capable of penetrating alveolar and gastric cells. Various natural lipids including phosphatidylcholines (PC), phosphatidylethanolamine (PE), sphingosine (So), ceramides (Cer), and fatty acid (FA), are abundant in decoctions of Traditional Chinese Medicine (TCM), such as *Lonicera japonica*, *Rhodiola crenulate*, *Andrographis paniculata*, and *Taraxacum mongolicum* [43,44,45]. Noteworthily, sphingosine, a key component of plant cell membranes, is widely distributed and contributes to physiological processes, such as cell signaling, proliferation, and apoptosis [46,47]. These findings provide an innovative therapeutic strategy for delivering oral exogenous sRNAs. Furthermore, Cao et al. [48] developed the “Bencao (herbal) sRNA Atlas” using high-throughput small RNA sequencing, identifying 20,758,257 unique sequences among 245 TCM decoctions. Bioinformatics approaches enable the comparison of plant-derived sRNAs sequences with mammalian genomes, facilitating the identification of therapeutic sRNA candidates. For instance, PGY-sRNA-6, which is *Taraxacum mongolicum*-derived, exhibits anti-inflammatory effects by targeting the v-Rel Avian Reticuloendotheliosis Viral Oncogene Homolog A (*RELA*) gene, a component of the nuclear factor kappa-B (NF-κB) transcription factor [49]. Similarly, HJT-sRNA-m7, isolated from *Rhodiola crenulate*, mitigates lung fibrosis by regulating three fibrotic proteins: alpha smooth muscle Actin (α-SMA), fibronectin, and collagen type III alpha 1 [49,50]. These candidate sRNAs can be chemically synthesized and validated in vitro using dual luciferase reporter assays to evaluate their regulation of disease-associated target genes. Following preclinical validation, sRNA candidates meeting drug development criteria may advance to clinical trials and, ultimately, new drug applications.

## 3. Delivery Systems of Plant-Derived sRNAs

### 3.1. Plant-Derived Exosomes

Plant-derived sRNAs, as natural bioactive macromolecules, exhibit limited in vivo bioavailability due to their instability and susceptibility to degradation in external environments, which restricts their clinical potential [51]. Recent studies have demonstrated that plant-derived exosomes, functioning as natural nanocarriers, effectively overcome these limitations [52]. These exosomes serve as an ideal delivery system for plant miRNAs and encapsulate a diverse array of active components, including nucleic acids, proteins, and lipid metabolites, some of which remain partially characterized [53]. Compared to synthetic liposomes, plant-derived exosomes offer distinct advantages, including low immunogenicity due to their natural origin, enabling safe passage across the intestinal barrier and targeted organ delivery. Their functional properties are comparable to those of mammalian-derived exosomes. Notably, plant-derived exosomes elicit negligible immune responses, highlighting their exceptional biosafety and biocompatibility [54]. These attributes position plant-derived exosomes as a promising drug delivery platform, particularly for bioactive macromolecules such as sRNAs and therapeutic proteins [53]. They hold significant potential in advanced therapeutic fields, including targeted cancer therapy and immunomodulation, paving the way for innovative drugs and delivery systems [55,56].

#### 3.1.1. Plant Exosome-like Nanoparticles (PENs)

Plant exosome-like nanoparticles (PENs), also termed “botanosomes”, range in size from 30 to 300 nm, which demonstrate the ability to deliver therapeutic agents in vivo [49]. PENs can be isolated from various fresh plants, including ginger, grapefruit, garlic, and *Houttuynia cordata* [57,58,59]. Owing to the diverse pharmacological properties of natural plants, PENs have emerged as a promising alternative to mammalian cell-derived exosomes, offering researchers a valuable tool to overcome the technical limitations associated with mammalian vesicles [60]. As a promising alternative drug delivery system, PENs are suitable for oral, intranasal, and intravenous administration, exhibiting excellent stability and biocompatibility [12,61]. Their unique physiological, chemical, biological properties, combined with their large-scale producibility, enable PENs to deliver diverse therapeutic payloads and hold substantial potential for disease therapy and nanocarriers development [62,63]. Ou et al. [12] identified a plant-derived exosome from *Catharanthus roseus* that serves as a stable nano-biomaterial, capable of resisting enzymatic digestion, extreme pH, and simulated gastrointestinal fluids, positioning it as a promising candidate for post-chemotherapy immune adjuvant therapy. Additionally, grapefruit-derived nanovectors efficiently deliver siRNA, DNA expression vectors, proteins, and chemotherapeutic agents to target disease sites [64]. Similarly, garlic exosome-like nanoparticles (GaELNs) are preferentially internalized by microglia, reducing brain inflammation in high-fat diet (HFD) mouse models [65].

#### 3.1.2. Plant Exosome-like Nanovesicles (PELNVs)

Plant exosome-like nanovesicles (PELNVs) are small, membrane-bound vesicles secreted by plant eukaryotic cells into the extracellular space [66]. These nanovesicles exhibit substantial therapeutic potential owing to their unique structure and composition. Specifically, PELNVs are enriched with bioactive components that confer diverse biological functions, including bioactive structural lipids with membrane-stabilizing properties that enhance cellular uptake, functional proteins enabling tissue-specific targeting, and cross-kingdom regulatory sRNA that precisely modulate gene expression in recipient cells [67]. These compositional attributes collectively impart PELNVs with excellent biocompatibility, robust stability in the gastrointestinal environment, and minimal immunogenicity [68]. Consequently, PELNVs combine intrinsic therapeutic activity with efficient delivery capabilities, positioning them as a promising next-generation multifunctional nanotherapeutic platform.

The biogenesis of PELNVs initiates with the formation of the trans-Golgi network or early endosome, which mature into multivesicular endosomes (MVEs) or multivesicular bodies (MVBs). Within MVBs, intraluminal vesicles (ILVs) selectively incorporate RNAs, lipids, and DNA. Subsequent fusion of MVBs with the plasma membrane releases PELNVs into the extracellular space [66,69]. PELNVs have emerged as promising delivery system for sRNA-based therapeutics, exhibiting cross-kingdom regulatory effects in mammals, including immunomodulation, anti-tumor activity, anti-inflammatory effects and tissue regeneration [14,66]. For example, ginger-derived exosome-like nanovesicles (GDENs), modified with folic acid (FA) as FA-3WJ/GDENs/siSurvivin, were delivered to xenograft KB cancer model, where they significantly suppressed *Survivin* expression in the tumor microenvironment [70]. Similarly, Ma et al. [71] developed *Panax ginseng* root-derived exosomes coated with neutrophil membranes and loaded with miRNA 182-5p, which mitigated acute lung injury by targeting the NADPH oxidase 4 (NOX4)/dynamin-related protein 1 (Drp-1)/NLR family pyrin domain containing 3 (NLRP3) signaling pathway. Furthermore, PELNVs demonstrate excellent biocompatibility and acid resistance. Cui et al. [72] engineered ginger-derived exosome-like nanovesicles (Exo@tac), modified with tetrahedral framework nucleic acids conjugated to antimicrobial peptides, which exhibited acid resistance. Oral administration of Exo@tac modulated intestinal flora in vitro and alleviated Parkinson’s disease symptoms in vivo via the microbial-gut-brain axis [72]. These findings underscore the potential of PELNVs as versatile nanotherapeutic platforms.

However, it is worth mentioning that current scientific consensus suggests that the therapeutic effects of plant-derived exosomes primarily arise from their complex bioactive cargo rather than solely from sRNAs. These exosomes, as multifaceted nanovesicles, encapsulate diverse proteins, lipids, and nucleic acid that collectively constitute their bioactivity. For instance, exosome-like nanoparticles from *Platycodon grandiflorum* contain membrane-associated proteins implicated in signal transduction and metabolic pathways [73]. Similarly, turmeric-derived exosome-like nanoparticles are enriched in lipids, proteins, and bioactive compound curcumin [74]. Therefore, future research should focus on systematically characterizing the active component profiles of plant-derived exosomes and elucidating their synergistic mechanisms of action. Such studies are essential for precisely defining the pharmacologically active constituents of these nanovesicles.

### 3.2. Herbal Decoctosome

**Figure 3:**
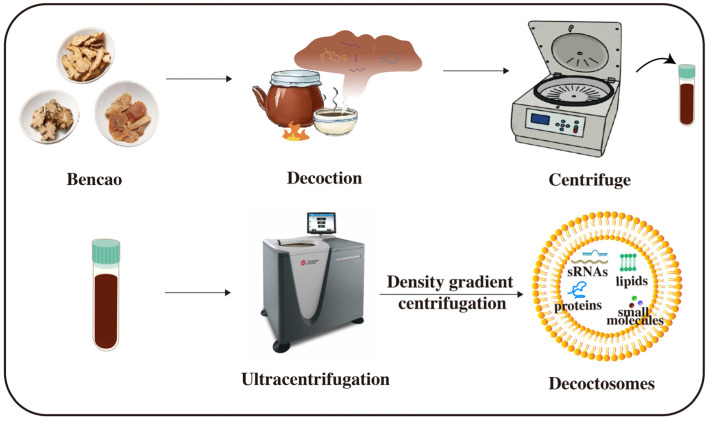
Extraction of herbal-derived sRNAs from decoctosomes. During TCM decoction, active herbal components, including sRNAs, lipids, and proteins, self-assemble into phospholipid-based nanoparticles through heat-induced processes. These decoctosomes facilitate the delivery of bioactive molecules into human cells, eliciting therapeutic effects.

The preparation of TCM decoctions, a standard traditional method, follows this process: precisely weighed TCM herbs are placed in a ceramic casserole, soaked in deionized water for 40 min, boiling for 30 min, simmering for 10 min, and filtering to remove residues [48]. During this process, heat-induced self-assembly of lipids and sRNAs forms stable nanoparticles termed “herbal decoctosomes” [6,48,49]. Upon oral administration, these plant-derived sRNAs assemble into decoctosomes, which protects them from gastrointestinal degradation, enabling their secretion into the bloodstream and delivery to mammalian tissues, including liver, lung, spleen, pancreas, and T cells [13,75]. Plant-derived phosphocholines further facilitate sRNA uptake by mammalian cells [49]. Herbal decoctosomes offer a novel drug delivery strategy, encapsulating active components such as sRNAs, lipids, and proteins within self-assembled phospholipid nanoparticles formed during decoction, thereby mediating therapeutic effects (Figure 3) [49,76]. For instance, compared to the decoction of *Rhodiola rosea* (Hong Jing Tian, HJT), the HJT decoctosome significantly reduced fibronectin expression in the MRC-5 cells, with the functional component HJT-sRNA-m7 identified as the key contributor [49]. Similarly, *Taraxacum mongolicum* (pugongying, PGY) decoctosomes exhibit superior anti-inflammatory activity compared to their decoction [49]. Relative to conventional TCM decoctions, herbal decoctosomes demonstrate enhanced therapeutic efficacy due to their naturally self-assembly, which encapsulates bioactive molecules, positioning them as promising delivery vehicles. Notably, HJT decoctosomes display anti-fibrotic potential in vivo by upregulating plasma levels of inflammatory cytokines and chemokines, such as IL-1α, IL-2, IL-5, IL-9, IL-10, IL-12 p40, IL-12 p70, IL-13, IL-17A, GM-CSF, IFN-γ, and MIP-1β in vivo [49].

### 3.3. Bencaosome

Bencaosomes are synthetic herbal decoctosomes engineered for medical applications, comprising sRNAs derived from TCM and plant-derived lipids, including sphinganine (d22:0), sphinganine (d18:1), sphinganine (d22:0), and LysoPA [49,50]. These components self-assemble into lipid nanoparticles with a membranous structure via water-bath heating. Optimal “herbal bencaosome” preparation involves thoroughly mixing lipids and sRNAs mimics, followed by heating in a water bath at 90 °C for 15 min to ensure coassembly of sRNAs with the lipid layer, thereby protecting sRNAs from degradation [49]. The concept of “herbal bencaosome” provides a novel framework for elucidating the mechanisms of sRNAs in TCM decoctions and expands the definition of bioactive TCM components beyond small molecules to encompass diverse classes of compounds. Recent studies demonstrate that the simplest bencaosomes, constructed from a single sRNA, exhibit pharmacological effects. Notable examples include BZL-sRNA-20 derived from *Scutellaria barbata* [6], PGY-sRNA-6 derived from *Taraxacum mongolicum* [49], HQi-sRNA-2 derived from *Scutellaria baicalensis* [8], XKC-sRNA-h3 derived from *Prunella vulgaris* L. [10], JGL-sRNA-h7 derived from *Gynostemma pentaphyllum* [11], XLGB28-sRNA from the Xianlinggubao formula [77], and TNF-α-sRNA-9 from the Sini decoction [78]. Furthermore, oral administration of “herbal Bencaosome” enhances patient compliance and reduces healthcare costs compared to injectable nucleic acid therapeutics, offering a convenient delivery platform [42].

### 3.4. Adeno-Associated Viruses (AAV)

Adeno-associated virus (AAV) vectors are highly efficient nucleic acid delivery platforms, evolved to target specific cell types and uniquely capable of actively delivering cargo to the nucleus [31]. These vectors are non-pathogenic, support sustained transgene expression, and demonstrate high transduction efficiency [79]. To date, the FDA has approved seven AAV-based gene therapy products for clinical use [11]. Notably, AAV vectors have been extensively utilized in strategies for delivering plant-derived exogenous miRNAs. For instance, Liu et al. [80] developed an AAV-based adenovirus expression vector encoding Cordyceps-derived miRNAs, miR1321, and miR3188 were transduced into HEK293 cells and administered via tail vein injection to treat of lung injury in preclinical models. As a robust nucleic acid delivery platform, AAV vectors efficiently deliver plant-derived sRNAs and transgenic therapeutics to specific target cells, achieving potent therapeutic effects.

## 4. The Pharmacological Activities of Plant-Derived sRNAs

**Figure 4:**
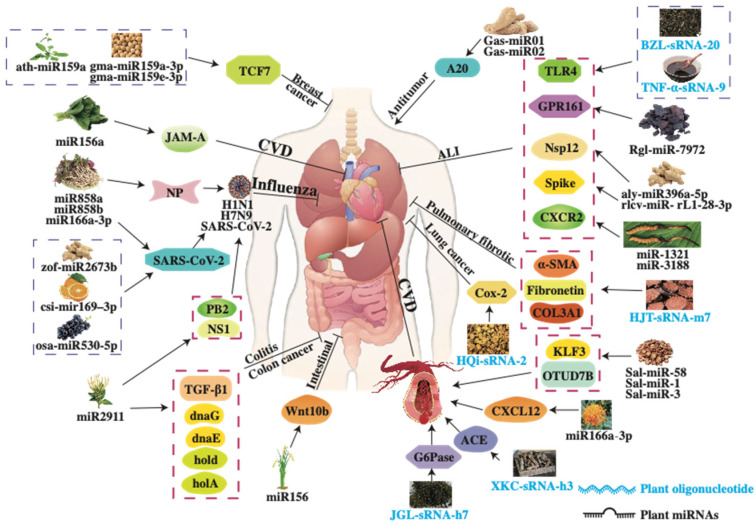
Regulatory mechanisms of medicinal plant-derived sRNAs in human diseases. Plant-derived sRNAs regulate gene expression through post-transcriptional silencing to mediate therapeutic effects in human diseases.

Plant-derived sRNAs have emerged as promising therapeutic tools for the treatment of diverse human diseases, including lung injury, influenza, hypertension, hyperglycemia, osteoporosis, skin disorders, inflammatory diseases and cancers [81] (Table 1 and Figure 4). However, the efficacy of plant-derived sRNAs still requires further validation in the coming years. Although extensive research has demonstrated the significant therapeutic potential of these sRNAs, the mechanisms governing their uptake and therapeutic effects in the human body remain poorly understood. Recent studies indicate that dietary plant-derived sRNAs are absorbed in mammals as exosomes or vesicular forms [14]. Following recognition by gastrointestinal epithelial cells, these sRNAs are internalized into gastric gland cells via the SIDT1 transporter [82]. Once inside, these vesicle-associated sRNAs may be taken by proximal stromal cells or lymphatic systems, ultimately targeting specific tissues [83]. Within target cells, sRNAs bind specific receptors, activate the RNAi pathway, and modulate downstream gene expression networks. However, the cross-kingdom regulatory effects of plant-derived sRNAs are influenced by factors such as sRNA stability, variability in sRNAs content across tissues of the same plant species, vesicle delivery efficiency, and targeting specificity [84]. The molecular mechanisms underlying these processes require further investigation.

### 4.1. Pulmonary Protective Activity

**Figure 5:**
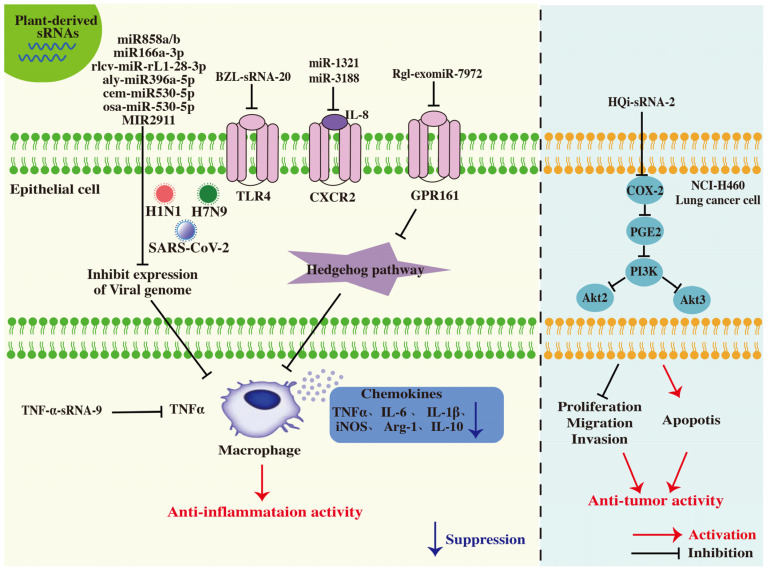
Molecular mechanisms of plant-derived sRNAs in pulmonary protection. Plant-derived sRNAs exert pulmonary protective effects by modulating immune responses and inhibiting lung cancer cells proliferation.

Numerous sRNAs derived from natural plants have demonstrated therapeutic potential in pulmonary disease. It is noteworthy that anti-inflammatory responses in pulmonary represent an effective strategy for treating acute lung injury (ALI) (Figure 5). Oral administration of bencaosome, a self-assembled nanoparticle composed of sphingosine (d22:0) and plant-derived sRNA (BZL-sRNA-20) from *Scutellaria barbata*, significantly alleviated lipopolysaccharide (LPS) and SARS-CoV-2-induced ALI in mice [6]. The mechanism underlying the protective effects of BZL-sRNA-20 involves the inhibition of pro-inflammatory cytokines via TLR4 expression, downregulation of the elevated pro-inflammatory cytokines levels induced by lipoteichoic acid (LTA) and polyinosinic-polycytidylic acid (Poly(I:C)), and prevention of cell death caused by infections with avian influenza virus H5N1, SARS-CoV-2.

Moreover, two oral delivery systems for *Rhodiola crenulata* have been constructed: the decoctosome and the bencaosome (composed of sphinganine d22:0 and HJT-sRNA-m7). Both systems exhibited anti-pulmonary fibrotic activity in TGF-β1-induced MRC-5 fibrotic cells, bleomycin-induced mice, and poly(I:C)-induced inflammatory mouse models [42,49]. Similarly, PGY-sRNA-6, derived from *Taraxacum mongolicum*, displayed anti-inflammatory effects by mitigating pulmonary inflammation. Continuous oral administration of sphinganine (d22:0)-PGY-sRNA-6 for one week significantly ameliorated poly(I:C)-induced pulmonary inflammation and reduced plasma levels of IL-1α, IL-5, and RANTES in mice [49]. Additionally, Rgl-exomiR-7972, the primary active component of exosome-like nanoparticles (ELNs) derived from fresh *Rehmanniae Radix*, exhibited superior protective effects against LPS-induced acute lung inflammation compared to the well-known chemical markers catalpol and acteoside [85]. Mechanistically, Rgl-exomiR-7972 attenuated lung inflammation by downregulating GPR161 expression, activating the Hedgehog signaling pathway, and inhibiting *Escherichia coli* biofilm formation by targeting the virulence gene *sxt2*, thereby restoring gut microbiota homeostasis. Ginger exosome-like nanoparticle (GELN) containing miRNA aly-miR396a-5p and rlcv-miR- rL1-28-3p represent a potential therapeutic agent for lung inflammation. These miRNAs mitigate SARS-CoV-2-induced cytopathic effects by downregulating Nsp12 expression [86]. Additionally, miR-1321 and miR-3188, derived from *Cordyceps militaris*, show therapeutic potential for ALI by directly targeting the 3’-UTR of *CXCR2* through an AAV vectors-mediated delivery system [80].

Acute respiratory distress syndrome (ARDS) is an acute, diffuse inflammatory condition resulting in lung injury [102]. Tumor necrosis factor-α (TNF-α), a pivotal inflammatory factor, plays a critical role in immune regulation and the initiation of inflammatory responses [103]. Sini decoction, a widely utilized TCM formulation, has been extensively validated for its therapeutic efficacy in treating ARDS in clinical practice [104,105]. Notably, Jiang et al. [78] employed bioinformatics analyses combined with pharmacological experiments to demonstrate that TNF-α-sRNA-9, an sRNA derived from Sini decoction, mitigates lung injury in a mouse model of mild ARDS by targeting the TNF-α signaling pathway.

Plant-derived sRNA have demonstrated potential in suppressing lung cancer progression. The oligonucleotide HQi-sRNA-2, derived from the medicinal plant *Scutellaria baicalensis*, exhibits promising therapeutic effects in a spontaneous lung cancer model [8]. Preclinical studies have confirmed that 3’-methylated HQi-sRNA-2 significantly inhibits the proliferation, migration, and invasion of NCI-H460 human lung cancer cells by targeting COX-2/PTGS2 and downregulating the PI3K/AKT signaling pathways. Oral administration of sphingosine (d18:1)-HQi-sRNA-2 bencaosome prolonged survival and reduced tumor burden in Kras^LSL-G12D^p53^fl/fl^ lung cancer mice. Compared to the positive control paclitaxel, HQi-sRNA-2 exhibits comparable efficacy with potentially lower toxicity [8].

### 4.2. Antiviral Activity

TCM displayed a significant role in epidemic prevention and control during the coronavirus disease 2019 (COVID-19) pandemic, offering substantial support in combating the outbreak [106,107]. Numerous studies have shown that miRNAs derived from medicinal plant, including honeysuckle, ginger, orange, and grapefruit, exhibit antiviral activity against SARS-CoV-2 through cross-kingdom regulation [82,94,95].

MIR2911, a honeysuckle-derived microRNA, exhibited broad antiviral activity against H1N1, H5N1, H7N9 and SARS-CoV-2 [9,82]. It effectively suppresses SARS-CoV-2 replication in COVID-19 patients, demonstrating significant therapeutic potential [82]. Using computational genomics, Mangukia [93,95] et al. identified a ginger-derived sRNA (zof-miR2673b) and an orange-derived sRNA (csi-mir169–3p), both targeting the conserved 3’ UTR of the SARS-CoV-2 genome. Additionally, cem-miR530-5p and osa-miR-530-5p, derived from edible nanoparticles (ENPs) of ginger and grapefruit, bind to the *ORF1ab* gene region of the SARS-CoV-2 genome [94]. Interestingly, SIDT1 protein, a nucleic acid transporter, mediates the cellular uptake of dietary miRNAs [13,82,108].

Clinical data from patients with COVID-19 treated with Toujie Quwen (TQ) granules revealed that peripheral blood mononuclear cells (PBMCs) isolated from their blood contained thousands of sRNA sequences derived from TCM, including *Radix Bupleuri* (Chai Hu), *Isatis indigotica* (Da Qing Ye), *Forsythia suspensa* (Lian Qiao), *S. baicalensis* (Huang Qin), and *Fritillaria thunbergii* (Zhe Bei Mu). Using miRanda algorithms, the top 30 novel increased sRNAs and the top 30 double increased sRNAs from TQ granules in PBMC samples were predicted to target approximately 80% of downregulated differentially expressed genes (DEGs) [44]. Pathway enrichment analysis demonstrated significant overlap between the downregulated DEGs and the predicted target genes of the most prevalent herbal sRNAs in the blood cells of these patients [44]. Moreover, validation in a Poly(I:C)-induced A549 cell inflammation model confirmed that the sRNAs derived from three effective TCM-*Citrus maxima* (Ju Hong), *Trichosanthes kirilowii* (Gua Lou Pi), and *Citrus medica* (Fo Shou)-suppressed signaling pathways associated with coronavirus infection [44].

Additionally, TCM-derived sRNAs have demonstrated antiviral activity against various viruses beyond SARS-CoV-2. Zhou et al. [9] identified an active miRNA, the honeysuckle-encoded atypical MIR2911, which directly targets the H1N1-encoded *PB2* and *NS1* genes, the H3N2-encoded *HA* gene, the H5N1-encoded *NP* gene, and the H7N9-encoded *HA* gene. Zhu et al. [59] reported that *Houttuynia cordata*-derived sRNAs, including miR858a, miR858b, and miR166a-3p, exhibit antiviral effects against respiratory RNA viruses when delivered via exosome-like nanoparticles. Specifically, miR858a and miR858b target the H1N1-endoced *NP* gene, while miR166a-3p targets the SARS-CoV-2-endoced *ORF1ab* gene. Furthermore, *p*-MIR858b, derived from *Moringa oleifera* seeds, was shown to target the HIV-1-encoded *VAV1* gene, reducing the expression of both VAV1 and p24 proteins [101].

### 4.3. Anti-Hypertension

*Prunella vulgaris* L. has been recognized as a commonly used herb in TCM decoctions for the management of hypertension [109]. Tang et al. [10] performed high-throughput sequencing of sRNAs from *P. vulgaris* decoction and the plasma of healthy individuals who consumed the decoction, identifying the 10 most abundant sRNAs. Notably, XKC-sRNA-h3 was shown to act as a natural antihypertensive agent by modulating ACE protein expression through cross-kingdom regulation. Further studies revealed that a sphingosine (d18:1)-XKC-sRNA-h3 constructed bencaosome targets the 3’-UTR of the *ACE* gene, suppressing ACE protein expression. Compared to captopril, this bencaosome significantly reduced serum creatinine and urea nitrogen levels in mice, demonstrating superior efficacy in alleviating hypertensive in vivo [10].

### 4.4. Anti-Hyperglycemia Activity

Diabetes mellitus, a complex metabolic disorder, poses a major global health challenge, driven by the obesity pandemic and the increasing prevalence of type 2 diabetes mellitus (T2DM) [110]. It is associated with severe complications, including cardiovascular disease, chronic kidney disease, neuropathy, and diabetic retinopathy [111,112,113]. *Gynostemma pentaphyllum* [Thunb.] Makino (Jiao Gu Lan, JGL) is a TCM widely recognized for its hypoglycemia effects [114]. Tang et al. [11] developed an orally administrated delivery system for therapeutic sRNA, sphingosine (d18:1)-JGL-sRNA-h7 bencaosomes. These nanoparticles effectively alleviated hyperglycemia in db/db mice and beagle dogs by targeting the 3’-UTR of *G6Pase* mRNA, thereby reducing G6Pase protein expression in the liver.

### 4.5. Anti-Osteoporosis Activity

Osteoporosis, a prevalent systemic metabolic bone disorder, is characterized by bone fragility and an increased risk of fractures [115]. In 2002, the Xianlinggubao (XLGB) capsule, a TCM formulation, was approved by the Chinese State Food and Drug Administration for the clinical management of osteoporosis [116]. Du et al. [77] identified 40 differentially expressed sRNA in human blood samples before and after XLGB capsule administration through high-throughput sequencing. Among them, the natural oligonucleotide XLGB28-sRNA was shown to suppress receptor activator of nuclear factor-κB ligand (RANKL) protein expression by selectively targeting and silencing the *TNFSF11* gene [77]. Inhibition of RANKL enhanced muscle strength and restored bone mass by suppressing osteoclasts formation and promoting osteoblasts differentiation, thereby improving bone mineralization [117]. Furthermore, oral administration of Lyso PA (16:0)-XLGB28-sRNA bencaosomes nanoparticles ameliorated bone loss in an estrogen deficiency-induced mouse model of osteoporosis [77].

### 4.6. Anti-Skin Aging Activity

Ultraviolet radiation is a primary environmental factor contributing to skin photoaging, characterized by impaired epidermal keratinization, abnormal hyperplasia, reduced collagen levels, loss of elasticity, and wrinkle formation [118,119]. Substantial evidence indicates that plant-derived exosome-like nanovesicles exert cross-kingdom regulatory effects through miRNAs to mitigate skin aging. For instance, nanovesicles derived from *Phellinus linteus* containing miR-CM1 suppress the expression of Mical2 via cross-kingdom regulation, thereby attenuating UV-induced skin aging [99]. Similarly, Choi et al. [120] demonstrated that exosome-like nanoparticles derived from ginseng roots alleviate UV- and oxidative stress-induced skin damage by downregulating mRNA levels of aging-related genes, Matrix Metalloproteinase (MMP) 2 and 3, proinflammatory genes, *COX-2* and Interleukin-6 (*IL-6*), and the cellular senescence biomarker p21. This effect is potentially mediated by the suppression of activator protein-1 signaling.

### 4.7. Anti-Inflammatory Activity

Inflammation treatment is another application of plant-sRNAs. *Curcuma longa*-derived clo-mir-14 exhibited stability in mammalian serum and target human genes associated with rheumatoid arthritis [121,122]. Similarly, Li et al. [90]. demonstrated that honeysuckle-derived MIR2911 ameliorated DSS-induced colitis in vivo by regulating intestinal microbiota, reducing *Escherichia-Shigella* abundance, and improving colitis symptoms. Additionally, *Salvia miltiorrhiza*-derived sal-miR-58 induces autophagy and attenuates inflammation in VSMCs through cross-species regulation of the KLF3/neural precursor cell-expressed developmentally downregulated 4-like (NEDD4L)/platelet isoform of phosphofructokinase (PFKP) signaling pathway [97]. Additionally, sal-miR-1 and sal-miR-3 derived from *S. miltiorrhiza* suppress thrombin-induced VSMC migration and monocyte adhesion by targeting the 3’UTR of *OUTD7B*, thereby blocking OUTD7B expression [98].

Furthermore, numerous pharmacology studies have explored the anti-inflammatory effects of miRNAs in ginger-derived exosome-like nanoparticles (GELNs). Teng et al. [123] reported that mdo-miR7267-3p, derived from GELN, is selectively taken up by *Lactobacillaceae* and targets genes in *Lactobacillus rhamnosus* to enhance indole-3-carboxaldehyde production, thereby ameliorating murine colitis via IL-22-dependent pathways. Similarly, Yin et al. also identified several GELN-derived miRNAs, including Novel_40, cca-miR156b, vvi-miR396a, ath-miR159a and gma-miR396h, which exhibit anti-inflammatory effects in LPS-stimulated Caco-2 cells by downregulating *NF-κB*, *IL-6*, *IL-8* and *TNF-α* gene expression [57].

Nanovesicles derived from *Carthamus tinctorius* L., containing miR166a-3p, exhibit therapeutic effects on atherosclerosis [96]. The underlying mechanism involves miR166a-3p exerting an anti-inflammatory effect on oxidized low-density lipoprotein-stimulated human umbilical vein endothelial cells (HUVECs). This effect is mediated by directly targeting the *CXCL12* gene, thereby downregulating the CXCL12 signaling pathway.

### 4.8. Anti-Tumor Activity

Numerous studies have demonstrated that herbal components can modulate the immune system and exert anti-tumor effects through nanomedicine-based drug delivery systems. Honeysuckle-derived miR2911 exhibits anti-tumor effects in colon cancer by targeting *TGF-β1* mRNA in tumor-bearing wild type *Sidt1*^+/+^ and nude type *Sidt1*^-/-^ mouse models [91]. This plant-derived miRNA induces *TGF-β1* gene silencing in targeted tissues, promotes T lymphocytes infiltration, and inhibits colon tumor development. Similarly, plant-derived miR159, including *Arabidopsis thaliana* ath-miR159a and *Glycine max* gma-miR159a-3p and gma-miR1593-3p, demonstrates anticancer activity against breast tumors in mammals [100]. Oral administration of miR159 mimic significantly suppresses the growth of xenograft breast tumors in vivo by targeting *TCF7*, a gene encoding a Wnt signaling transcription factor, resulting in reduced MYC protein levels [100]. Additionally, elevated A20 expression has been associated with various solid human tumors [124]. *Gastrodia elata* Blume-derived sRNAs, Gas-miR01 and Gas-miR02, were shown to target and suppress *A20* gene expression in 293T cells [92]. These findings may elucidate the complex mechanisms underlying A20’s role in tumor development and treatment response.

## 5. The Advantages of Plant-Derived sRNA Therapy

Small molecule drugs and multi-component formulations derived from plants are widely utilized in medical field [125]. However, these agents have limitations, including adverse effects and uncertainties surrounding drug-drug interaction [126,127]. In contrast, plant-derived sRNAs regulate genes expression by selectively targeting and degrading diseases-associated mRNAs, enabling precision therapeutics [128]. This unique mechanism, characterized by its natural origin and high selectivity, may provide superior efficacy and safety compared to conventional pharmaceuticals. Following decades of extensive research and advancements in nucleic acid platform technologies, plant-derived sRNA therapeutics have demonstrated significant advantages in targeting precision, efficacy, and safety [88,129]. These developments underscore their potential for future applications in personalized medicine and precision healthcare.

### 5.1. Minor Adverse Effect

Many nuclear acid-based therapeutics, like oligonucleotide, currently in development or on the market, are synthetically designed. However, these drugs have encountered setbacks due to adverse reactions. For example, the antisense oligonucleotide Mipomersen, used to treat hypercholesterolemia, was withdrawn from the market due to adverse effects, including angioedema [130]. In contrast, plant-derived sRNAs are sourced from natural plants species, and typically exhibit good biocompatibility with the human biological system, reducing the risk of immune rejection and toxicity [10]. Compared to synthetic sRNA therapeutics, plant-derived sRNAs are associated with fewer adverse reactions, likely due to their long-term evolutionary adaptation in natural environments, which minimizes harm to the human body.

### 5.2. Better Patient Compliance

The development of orally administered conventional small-molecule therapeutics faces significant challenges due to physiological barriers in the gastrointestinal tract and the first-pass effect in liver [131,132]. However, naturally plant-derived mature miRNAs are efficiently absorbed through the mammalian gastrointestinal tract following oral administration; they regulate the host gene expression in vivo, offering a promising platform for next-generation RNAi therapeutic strategies [88,100,133]. Specifically, 3-methylated plant-derived mature miRNAs are taken up mammalian gastrointestinal gland cells via SIDT1, which facilitates the absorption of exogenous miRNAs [13]. Theses miRNAs demonstrate remarkable stability in gastric fluid at pH 2, and after entering the mammalian circulatory, remain stably packaged within MVs and exosomes in blood and various tissues [108,134]. In addition, plant-derived sRNAs can self-assemble into decoctosomes by combining with plant-derived lipid sphingosine during thermal processing [48,49]. This delivery platform protects exogenous sRNAs from degradation in vivo and enhances their delivery efficiency [48,50]. Consequently, oral administration of plant-derived sRNAs improves patient compliance and reduces treatment costs, providing a novel perspective for the developing of natural plant-based small nucleic acids therapeutics.

### 5.3. Enhanced Targeting and Specificity

Numerous therapeutic drugs approved by regulatory agencies such as the FDA, EMA, and NMPA are single components therapies, including small molecules, proteins, antibodies, and oligonucleotide-based therapies. These drugs target one or more pathogenic genes to address specific indications. However, drug–drug interactions may reduce therapeutic efficacy or cause adverse effects. sRNAs therapies offer a promising alternative by regulating gene expression at the post-transcriptional level with high precision and specificity. sRNA therapies target pathogenic gene mRNAs with single-nucleotide resolution, enabling upstream modulation of gene expression [135]. The specificity and targeting precision of small nucleic acid drugs are central to their therapeutic efficacy and safety, allowing selective inhibition of disease-related genes while minimizing off-target effects on unrelated genes or biological pathways [136]. This targeted approach modulates specific molecular pathways without disrupting non-disease-related cellular processes, enhancing both efficacy and safety.

## 6. Limitations and Prospects

Although plant-derived sRNAs exhibit promising therapeutic potential, their practical application faces several critical challenges: (1) Current technologies for plant-derived sRNAs isolation and purification struggle to meet the demand for high-purity, large-scale sRNAs, significantly hindering subsequent studies; (2) The differences in the compositional and function of sRNAs in different plant varieties may significantly impact the stability of their biological activity and the reproducibility of clinical outcomes; (3) Plant-derived sRNAs are abundant and target multiple genes. However, the precise role of total miRNA in Chinese herbal medicine is not fully understood, and its mechanisms of action require further exploration through extensive basic research; (4) There is no clear consensus on the standardized extraction methods or quality control for small nucleic acids extracted from traditional Chinese medicine decoctions; (5) The entire production chain—from plant cultivation to final product formulation—faces technical challenges, including the development of efficient extraction methods, cost control of raw materials, and standardization of cultivation practices; (6) There is a lack of high-quality clinical research. Currently, the findings from most research on plant-derived sRNAs have not been applied in clinical studies. In conclusion, overcoming these challenges demands interdisciplinary collaboration, integrating expertise from multi-omics, nanomedicine, and clinical research to facilitate the translation of plant-derived sRNAs from laboratory studies to clinical applications.

Plant-derived sRNAs may regulate human gene expression and influence the development and progression of diseases [100,137]. However, evidence supporting the role of dietary sRNAs in human health and disease remains limited. Future research on plant small nucleic acids should further investigate the absorption of plant miRNAs from food and their physiological effects in animal models. Additionally, evaluating the in vivo uptake of sRNAs from diverse plant sources will provide evidence for sRNA-mediated cross-kingdom interactions, offering novel insights into the etiology, prevention, and treatment of human diseases. Exosome and nanovesicles demonstrate targeted specificity for recipient cells and exhibit therapeutic effects in vivo, underscoring their potential as natural delivery systems for drug delivery applications. However, simple chemical modifications or delivery methods for exogenous plant-derived sRNAs faced significant challenges, including limited efficacy and specificity. Future research should focus on optimizing chemical modifications and advanced delivery systems to enhance safety and efficacy, endosomal escape, and tissue-specific targeting while minimizing side effects. Combining nucleic acid modifications with innovative delivery platforms could improve therapeutic outcomes, although this approach may increase technical complexity and costs associated with clinical translation.

## 7. Conclusions

In conclusion, this review systematically summarized the classification, biosynthetic pathways, and gene-silencing mechanisms of plant-derived sRNAs. We comprehensively analyzed cross-kingdom delivery strategies mediated by various nanocarriers and elucidate the therapeutic effects and molecular mechanisms of plant-derived sRNAs in diverse disease. Additionally, we objectively discussed their advantages and current limitations. Although research on plant-derived sRNAs is advancing rapidly, with preliminary evidence supporting their therapeutic potential, further in-depth studies are critical to enable their broad application in precision medicine.

## Figures and Tables

**Table 1:** Functions of plant-derived sRNAs.

sRNAs	Origin	Delivery System	Therapeutic Disease	Target Gene	Model	Refs.
BZL-sRNA-20	*Scutellaria barbata*	Bencaosome	Acute lung injury	Toll-like receptor 4 (*TLR4*)	In vitro: LPS-induced THP-1 cell model, lipoteichoic acid (LTA)-induced U937 cell model and polyinosinic-polycytidylic acid (poly(I:C))-induced A549 cells; H5N1 virus-infected A549 cell model, SARS-CoV-2 infected Vero E6 cell model. In vivo: LPS and SARS-CoV-2-induced acute lung injury C57BL/6J mice models.	[6]
HJT-sRNA-m7	*Rhodiola crenulata*	Liposome	Pulmonary fibrotic	*α-SMA*, *fibronetin*, and *COL3A1*	In vivo: bleomycin-induced pulmonary fibrosis model in C57BL/6J mice.	[42]
PGY-sRNA-6	*Taraxacum mongolicum*	Bencaosome and decoctosome	Inflammatory	*RELA*	In vitro: poly(I:C)-induced A549 cell; In vivo: poly(I:C)-induced inflammatory model in C57BL/6J mice.	[49]
Rgl-exomiR-7972	*Rehmanniae Radix*	Exosome	Acute lung injury (ALI)	G protein-coupled receptor 161 (*GPR161*)	In vitro: LPS-induced RAW264.7 cell; In vivo: LPS-induced acute lung inflammation model in BALB/c mice.	[85]
TNF-α-sRNA-9	Sini decoction	Bencaosome	Acute lung injury	*TLR4*	In vitro: LTA-induced U937 cell, poly(I:C)-induced A549 cell, and Vero E6 infected with SARS-CoV-2; In vivo: LPS-induced acute lung injury model in C57BL/6J mice.	[78]
HQi-sRNA-2	*Scutellaria baicalensis*	Bencaosome	Lung cancer	Cyclooxygenase-2 (*COX-2*)	In vivo: *Kras*^LSL-G12D^*p53*^fl/fl^ lung cancer mice model.	[8]
aly-miR396a-5p	Ginger	Exosome-like nanoparticle	Lung inflammation	Nonstructural protein 12 (*Nsp12*)	In vivo: transfected with SARS-CoV-2 plasmids administered to C57BL/6 mice; In vitro: Vero E2 cells infected with SARS-CoV-2.	[86]
rlcv-miR- rL1-28-3p	Ginger	Exosome-like nanoparticle	Lung inflammation	*Spike*	In vitro: Vero E2 cells infected with SARS-CoV-2.	[86]
miR156	Crops	-	Intestinal cell proliferation	*Wnt10b*	In vitro: procine jejunum epithelial (IPEC-J2) cell	[87]
MIR156a	Cabbage, spinach, lettuce	Exosome	Cardiovascular disease (CVD)	Junction adhesion molecule-A (*JAM-A*)	In vitro: HAEC cell	[88]
MIR168a	Rice	Microvesicle	-	*LDLRAP1*	In vivo: C57BL/6J mice.	[7]
miR2911	Honeysuckle	Cell-derived microvesicle	Influenza A viruses	H1N1-encoded *PB2* and *NS1* genes	In vitro: H1N1 virus in Madin-Darby Cannine Kidney (MDCK) cells In vivo: H1N1, H5N1 and H7N9 influenza viruses inoculated BALB/c mice models.	[9]
MIR2911	Honeysuckle	Exosome	SARS-CoV-2	SARS-CoV-2 genome	In vitro: SARS-CoV-2 propagated in Vero E6 cells In vivo: moderate type patient infected by SARS-CoV-2 virus.	[82,89]
MIR2911	Honeysuckle	Small extracellular vesicles	colitis	*holA*, *holD*, *dnaE*, *dnaG* and *ligB*	In vivo: DSS-induced colitis model in C57BL/6J mice	[90]
miR2911	Honeysuckle	-	Colon cancer	Transforming Growth Factor-β1 (*TGF-β1*)	In vivo: tumor-bearing *Sidt1*^+/+^ and *Sidt1*^-/-^ mice models.	[91]
Gas-miR01/Gas-miR02	*Gastrodia elata* Blume	-	Tumor	*A20*	In vivo: 293T cells	[92]
Novel_40/cca-miR156b/vvi-miR396a/ath-miR159a/gma-miR396h	Ginger	Exosome-like nanoparticles	Inflammatory	-	In vitro: LPS-induced inflammation model in Caco-2 cell.	[57]
zof-miR2673b	*Zingiber officinale*	-	-	SARS-CoV-2 genome	-	[93]
Cme/osa-miR530-5p	Ginger and grapefruit	Edible nanoparticles (ENPs)	SARS-CoV-2	SARS- CoV-2 genome	-	[94]
csi-mir169–3p	*Citrus* *sinensis*	-	-	SARS-CoV-2 genome	-	[95]
miR166a-3p	*Carthamus tinctorius* L.	Nanovesicles	Atherosclerosis	Chemokine ligand 12 (*CXCL12*)	In vitro: ox-LDL-induced inflammation model in HUVECs In vivo: *ApoE*^-/-^ mice	[96]
Sal-miR-58	*Salvia miltiorrhiza*	-	Abdominal aortic aneurysm (AAA)	Kruppel-like factor 3 (*KLF3*)	In vitro: Ang II-induced mouse vascular smooth muscle cells (VSMCs); In vivo: Ang II-induced AAA model in *ApoE*^-/-^ mice.	[97]
Sal-miR-1/Sal-miR-3	*Salvia miltiorrhiza*	F-127 pluronic gel	Vascular remodel induced by vascular injury	OTU deubiquitinase 7B (*OTUD7B*)	In vitro: thrombin-induced the migration of VSMCs and monocyte adhesion to VSMCs; In vivo: intimal hyperplasia induced by carotid artery ligation in C57BL/6 mice.	[98]
miR-1321/miR-3188	*Cordyceps militaris*	Adeno-associated viruses (AAV)	Acute lung injury (ALI)	CXC-chemokine receptor 2 (*CXCR2*)	In vivo: bleomycin-induced ALI model in Balb/c mice	[80]
miR858a/miR858b	*Houttuynia cordata*	Exosome-Like Nanoparticles	RNA Viruses	*NP* gene in H1N1	-	[59]
miR166a-3p	*Houttuynia cordata*	Exosome-Like Nanoparticles	RNA Viruses	*ORF1ab* gene in SARS-CoV-2	-	[59]
XKC-sRNA-h3	*Prunella vulgaris* L.	Becaosome	Hypertension	*ACE*	In vivo: Ang II-induced hypertensive cardiac damage C57BL/6J mice model.	[10]
JGL-sRNA-h7	*Gynostemma pentaphyllum* [Thunb.] Makino	Bencaosome	Hyperglycemia	glucose-6-phosphatase (*G6Pase*)	In vivo: hyperglycemia in db/db mice model and beagle dogs’ model.	[11]
XLGB28-sRNA	Xianlinggubao (XLGB) formula	Bencaosome	Osteoporosis	tumor necrosis factor superfamily member 11 (*TNFSF11*)	In vitro: ascorbic acid and β-glycerol phosphate induced osteogenic MC3T3-E1 cells model. In vivo: estrogen deficiency-induced osteoporosis in C57BL/6J mice model.	[77]
miR-CM1	*Phellinus linteus*	Exosome-like nanovesicle	Ultraviolet-induced skin aging	Microtubule-Associated Protein-Lysine N-Methyltransferase 2 (*Mical2*), Dual-specificity phosphatase 18 (*DUSP18*), GRB2-related adaptor protein (*GRAP*) and RNA polymerase I transcription factor (*RRN3*)	In vitro: UV-induced HaCaT cell model. In vivo: UV-induced skin photoaging Kunming mice model.	[99]
ath-miR159a	*Arabidopsis thaliana*	-	Breast cancer	Transcription factor 7 (*TCF7*)	In vivo: MDA-MB-231 xenograft tumor induced NOD-*scid* IL2Rg^null^ (NSG) mice model.	[100]
gma-miR159a-3p/gma-miR159e-3p	*Glycine max*	-	Breast cancer	*TCF7*	In vivo: MDA-MB-231 xenograft tumor induced NOD-*scid* IL2Rg^null^ (NSG) mice model.	[100]
*p*-miR858b	*Moringa oleifera*	-	HIV infection	Vav1 oncogene (*VAV1*)	In vitro: HIV-infected PBMCs cell	[101]
